# Corrosion Risk to Metal-Based Artefacts in a Scientific and Technical Museum: An Assessment of Environmental and Exhibition Conditions

**DOI:** 10.3390/ma16124239

**Published:** 2023-06-08

**Authors:** María Teresa Molina, Emilio Cano, Irene Llorente, Blanca Ramírez-Barat

**Affiliations:** Centro Nacional de Investigaciones Metalúrgicas (CENIM), Consejo Superior de Investigaciones Científicas (CSIC), 28040 Madrid, Spain

**Keywords:** metal heritage, scientific-technical artefact, indoor corrosivity, ISO 11844, pollutants, organic acids, humidity

## Abstract

Materials such as wood, textiles, or plastics that are part of the exhibition system in museums are known to emit pollutants such as organic acids. Scientific and technical objects that include these materials in their composition can themselves be a potential source of emissions, which, together with inappropriate humidity and temperature conditions, can lead to corrosion of the metallic parts. In this work, we have studied the corrosivity of different locations in two venues of the Spanish National Museum of Science and Technology (MUNCYT). Coupons of the most representative metals from the collection were placed in different showcases and rooms for 9 months. The corrosion of the coupons has been evaluated in terms of the rate of mass gain, colour changes and characterisation of the corrosion products. The results were correlated to the relative humidity and concentration of gaseous pollutants to determine which metals are most susceptible to corrosion. The results show that metal artefacts exposed in showcases have a higher risk of corrosion than those exposed directly in the room, and that some pollutants are emitted by the artefacts. The corrosivity of the museum environment is low for copper, brass, and aluminium in most locations; however, some placements present a higher aggressivity for steel and lead, due to the high humidity and the presence of organic acids.

## 1. Introduction

Considering the value and irreplaceable character of cultural heritage, museums aim to achieve the best possible environmental conditions to ensure the long-term conservation of heritage assets. However, for many reasons, including economic, environmental and practical ones, it is quite common that optimal conditions are not achieved. Atmospheric pollutants (such as SO_2_, NO_x_, O_3_, sulphides and organic acids) are one of the main agents of degradation in museum collections [1]. Among these compounds, acetic and formic acids are the predominant pollutants in indoor atmospheres, as they originate from the emissions of materials used in the construction of showcases, exhibitions and storage, such as wood, paintings or adhesives [2,3]. The effect that these pollutants have on different heritage materials, as well as their exposure limits, has been extensively studied in recent decades [4,5,6], and considered by museums in their preventive conservation strategies. In collections made up of artefacts of the same material, controlling their exposure to aggressive environments and defining the optimal environmental conditions (humidity, temperature and illumination) to prevent their degradation is not usually a challenge. However, there are more complex collections wherein preventive conservation is a challenge, such as those of scientific and technical (ST) artefacts.

One of the peculiarities of scientific and technical heritage is that the same artefact can be made of several materials; metal, as the main material, is usually accompanied by wood, glass, paper, plastic, etc. [7,8]. Additionally, it can also contain different substances related to its functioning, such as lubricants, oils or dry chemical powders [9,10]. This multi-materiality may cause incompatibilities in the same artefact, and be a potential source of harmful emissions, affecting both itself and other objects exposed in the same space. In particular, metal-based artefacts are affected by the presence of these pollutants, relative humidity, and temperature, which cause their corrosion [2,11,12].

Analysis of VOC emissions can help in assessing the risks and understand the degradation processes of collections. In recent decades, several methods have been employed to detect and quantify the pollution of the indoor environment in museums and exhibitions, using in situ analytical techniques based on microextraction and chromatographic analysis of VOCs [13,14], as well as passive sampling devices (PSD) [1]. However, these alternatives can sometimes be very costly, and do not determine how harmful the detected contaminants may be to artefacts. Another approach is the use of metal coupons that act as corrosion dosimeters, based on the sensitivity of some metals to certain contaminants, e.g., silver to sulphides and lead to organic acids. This methodology is used to perform Oddy tests to assess the suitability of materials used in display cases and/or the storage of collections [15,16,17], or to carry out an assessment and classification of the corrosivity of the indoor environment based on ISO 11844 standards [18,19]. The combination of these alternatives results in a complete analysis of the exposure environment and the degree of corrosion that metals undergo when exposed to it.

Despite the complexity of conservation that ST objects are subjected to, there are no studies that have investigated the environment and risks of degradation of this type of collection; said studies would help museums to take preventive conservation measures. Therefore, the main objective of this work is to assess the pollution and to investigate how and to what extent the different environmental and exhibition conditions of scientific-technical collections can affect the corrosion and therefore the conservation of metal-based artefacts. For this purpose, a study was carried out in the National Museum of Science and Technology of Spain (MUNCYT), which has approximately 19,000 artefacts, including scientific instruments, vehicles, machines, and industrial tools [20].

## 2. Materials and Methods

Lead, copper, aluminium, carbon steel, and brass were selected as the most representative metals in this collection [21], and coupons acting as metal dosimeters, were prepared based on the methodology of ISO 11844-2 [19]. These coupons are used to evaluate the corrosion experienced by each metal after its exposure in different locations of the museum (showcases and rooms) with different methods: measuring the rate of mass increase and classifying the corrosivity of the environment for each metal, evaluating the colour changes experienced, and characterising the corrosion products formed on the coupons by XRD. The results are compared and evaluated in terms of the environmental and pollution data measured at the different locations, according to [22].

### 2.1. Museums Locations

**Figure 1:**
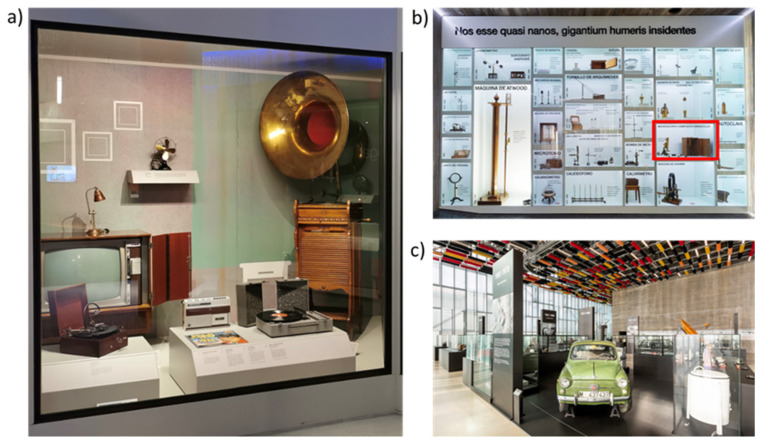
(**a**) Home, Sweet Home (S2) showcase of MUNCYT Alcobendas. (**b**) Ex cathedra showcase (S6). The sub-vitrine where the rack with the metallic dosimeters was placed is marked in red. (**c**) XX Century room (R7) of MUNCYT A Coruña.

MUNCYT is a decentralised museum with venues in A Coruña (Galicia, Spain) and Alcobendas (Madrid, Spain). The atmospheric conditions of these two cities are very different from each other; MUNCYT A Coruña is located in the northwest of Spain in a city with mild temperatures and a rainy climate, less than 200 metres from the coast, and the Alcobendas venue is in the centre of the country, close to Madrid, at about 700 m above sea level, in an area with more extreme temperatures and lower precipitation. As a result, the climatic and atmospheric conditions outside the museum start from a different point, and are expected to be much more humid and saline at A Coruña venue. In both venues, the ST collection includes a wide variety of artefacts of different compositions and dimensions, which are exhibited using different systems. For this study, two display cases and two rooms have been selected at each venue:

1. MUNCYT Alcobendas
  - **Space–Time showcase (S1)**: This showcase’s large size extends over an entire room. It contains more than a hundred instruments for navigation, astronomy, topography, etc. It is the location with the greatest variety of material exhibited as a whole, with no separations between the artefacts.
  - **Home, Sweet Home showcase (S2)**: This showcase holds TVs, radio cassettes, telephones, irons, toys, etc., representing the experience of the home through utility and leisure after the end of the Second World War (Figure 1a). Wood, plastic, and brass and steel as metals predominate [21].
  - **Cabinet room (R3)**: This room comprises different showcases, with no ST artefacts outside them.
  - **Wheels room (R4)**: In contrast to the previous room, this room exhibits a large collection of cars, motorbikes, bicycles, and carriages. As a result, there is an abundance of tyres, lubricating oils, and plastics.
2. MUNCYT A Coruña
  - **Heritage showcase (S5)**: This showcase is similar to Space–Time, but is divided into several independent parts. The part containing three artefacts is selected, and glass, wood, and copper alloys are identified as the materials composing the artefacts.
  - **Ex cathedra showcase (S6)**: This showcase is one of the most complex, as it is divided into several sections, as can be seen in Figure 1b. It contains 19th century physics and chemistry laboratory artefacts made of wood, steel, copper, and brass. For the study, a compartment is selected, in which a binocular microscope and a wooden box of lenses are on display.
  - **XX Century room (R7)**: This room is large and is connected to other spaces. It is located in an open area of the museum, which has a glass enclosure system forming the perimeter structure of the building. It contains artefacts made of different materials, both inside and outside showcases (Figure 1c).
  - **Entrance room (R8)**: This is the museum’s visitor reception room, where the doors to the outside are open constantly. Hanging from the ceiling is an aluminium Midget Mustang aircraft from the late 1960s. There is also a showcase with temporarily changing artefacts.

### 2.2. Environmental Conditions: Pollutants, Humidity and Temperature

Numerous passive sampling devices are currently available on the market to quantify gaseous pollutants inside museums [1]. PSDs from IVL Swedish Environmental Research Institute Ltd., Göteborg, Sweden have been used for this study. These devices are based on the diffusion of air pollutants into an absorption medium–cellulose filter, which is then analysed, and in which the sorbed amount must be proportional to the ambient concentration of the gas [23]. One device for H_2_S (concentration range: 0.1–200 μg/m^3^) and one for SO_3_ (0.1–100 μg/m^3^), HF (0.2–40 μg/m^3^), HCl (0.3–100 μg/m^3^), HCOOH (1.5–200 μg/m^3^), and CH_3_COOH (1.5–250 μg/m^3^) were placed at all locations (except R3, which due to the uniformity of the exhibition space is very similar to R4). The exposure time was one month, according to the supplier’s recommendations; then, the PSD was sent to the laboratory for analysis and interpretation.

The conditions of relative humidity and temperature were recorded, during the 9 months for which the coupons were exposed, using a USB data logger model EL-USB-2 from EasyLog, Lascar electronics, Whiteparish, UK. The recorded data were averaged, identifying the maximum and minimum peak of the two variables. The monitored locations were the same as the PSDs. However, in R8, data were lost due to the loss of all devices.

### 2.3. Sample Preparation: Coupons and Racks

**Figure 2:**
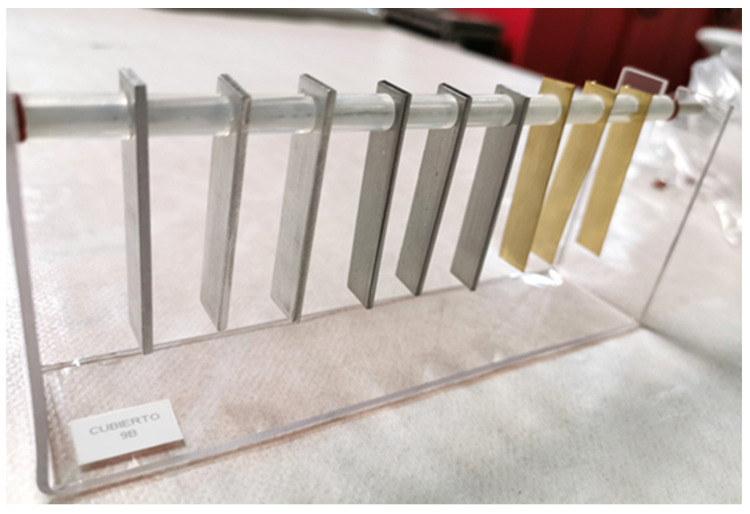
Rack model used for the exposure of the metal coupons. Aluminium, steel, and brass coupons are shown in this rack from left to right.

Coupons were prepared using a methodology based on ISO 11844-2 [19], and 10 mm × 50 mm coupons of variable thickness were prepared from metal sheets of different thickness: lead (0.5 mm), copper (0.25 mm), aluminium (2 mm), steel (2 mm), and brass (0.3 mm). The lead and copper are 99.9% pure, the steel a low carbon alloy (0.026% C, 0.15% Mn, 0.014% P and 0.026% S by weight), aluminium a 2024 alloy, and brass a CDA 260 alloy. Coupons were sanded, degreased and prepared in triplicate according to [19]. For exposure, a 4.5 mm diameter hole was drilled in the coupons to hang them with a fibreglass rod in a PET (Veralite^®^, IPB, Waregem, Belgium) rack, with a 10 mm gap between each coupon to ensure air circulation (Figure 2). The racks were placed in the eight locations described in Section 2.1 for 9 months (from September to July).

### 2.4. Calculation of the Rate of Mass Increase

ISO 11844-2 proposes several methods to determine the corrosion rate of exposed coupons. Among them, the mass variation method is one of the most widely used because of its fast and easy use. The rate of mass increase depends mainly on the elements from the contaminants reacting and adhering to the metal surface. Some studies opt for the rate of mass loss [24] or the electrochemical reduction of corrosion products [25] to assess the corrosivity of the locations, but this can also have disadvantages, such as the overestimation of low corrosion rates through uncontrolled stripping of the metal, or underestimation of corrosion through reducing metals with soluble corrosion products such as lead [16]. In view of these uncertainties, we decided to consider only the mass gain of the coupons, and to consider the result a semi-quantification of corrosion in order to compare it to the colour change and corrosion products.

For this purpose, each coupon was weighed twice on a microbalance with an accuracy of ±0.1 mg, in relation to a stainless steel standard of a mass slightly higher than the coupon. The difference between the first mass of the coupon *m*_1_ and that of the reference standard *m_r_*_,1_ is calculated as (*m_r_*_,1_ − *m*_1_), and the second one as (*m_r_*_,2_ *− m*_2_). The mass of the coupons is calculated with respect to the reference coupon as the average of the differences (*m*) in mg, as indicated in Equation (1):

(1) $$m=\frac{\left(m_{r,1}-m_{1}\right)+\left(m_{r,2}-m_{2}\right)}{2}$$

This results in *m_be_*, which corresponds to the mass of the coupons before exposure, and *m_ae_,* which corresponds to the mass of the coupons after exposure. The rate of mass increase (*r_mi_*) in mg·m^−2^·a^−1^ for each metal coupon is calculated from Equation (2), where *A* is the coupon area in m^2^ and *t* is the exposure time in years, represented by unit symbol *a*.

(2) $$r_{mi}=\frac{m_{ae}-m_{be}}{A\cdot t}$$

For the environment corrosivity classification according to the standard, only steel and copper can be considered in this study (Table 1) (lead is not yet included in the mass increase in the latest version). For brass, the values for copper and zinc are taken as a reference to establish a classification proposal, according to the results of this study.

### 2.5. Characterisation Techniques

Colour measurements were made with a CM-700d spectrophotometer, Konica-Minolta, Ramsey, NJ, USA, using the 1976 CIELAB colour space. The standard illuminant was D65, and the observer was set at 10°. The colour coordinates L*, a* and b* were recorded at six areas on the coupons (Ø = 6 mm), three on each side, before and after exposure. As the coupons are prepared in triplicate, an average is taken for each coupon to obtain the L*a*b* values. The colour differences were calculated from Equation (3):

(3) $$\Delta E=\sqrt{{\left(\Delta L^{*}\right)}^{2}+{\left(\Delta a^{*}\right)}^{2}+{\left(\Delta b^{*}\right)}^{2}}$$

XRD measurements were performed using a Bruker AXS D8 advance diffractometer (Bruker AXS GmbH, Karlsruhe, Germany) with Co radiation (λ = 1.78897 Å), equipped with a Goebel mirror and a LynxEye linear position-sensitive detector for ultrafast XRD measurements. In order to characterise the patinas formed with the least contribution of the substrate, XRD measurements were carried out in a grazing incidence condition (GIXRD) over a 2θ range, from 10° to 110°, with a step width of 0.03° and a counting time of 3.5 s per step. Qualitative identification of the crystalline phases present in the patinas formed was carried out with XRD patterns, using the JCPDS database and software.

## 3. Results and Discussion

### 3.1. Environmental Conditions

Relative humidity is a key condition in the corrosion of metals. For an ideal conservation of metallic heritage, RH should be as low as possible. However, low RH can cause damage in organic materials, and therefore is not acceptable in a mixed collection of these characteristics. In these situations, an RH between 35% and 55% and a temperature between 16 °C and 25 °C with controlled oscillations are recommended [26,27]. Table 2 shows how the RH and T data differ between the two museum venues, as expected. In locations S1, S2, and R4 of MUNCYT Alcobendas, the average RH value is good, and only in the room were maximum peaks above 55% reached. The high levels of humidity in showcases S5 and S6 at the A Coruña venue (and especially in the room R7) stand out, so it was expected that the coupons would suffer corrosion during their exposure.

In the same table, the concentration of pollutants from the PSDs is presented. Acetic (CH_3_COOH) and formic (HCOOH) acids were identified among the highest levels of contaminants, especially in showcases (S1, S2, S5 and S6). The degradation of some organic materials that compose the ST objects, the presence of wood (either on the objects or as part of the showcase) paints, and adhesives are the main source of emissions [6] that are concentrated in an enclosed space with little ventilation (in relation to the rooms). A high level of H_2_S was also detected, which can be produced by the degradation of the proteins in materials of animal origin such as wool, silk, or leather, or emitted by visitors [4]. The concentration of organic acids and sulphides is inversely correlated with the size of the space in which the objects are kept: higher concentrations were measured in smaller display cases S1 and S5, followed by the bigger cases S2 and S6, followed by the exhibition rooms (R4 and R7). The latter is highly ventilated and beside the glass walls of the building, therefore presenting very low concentrations of pollutants of indoor origin. On the other hand, it is the only area showing SO_2_ concentrations above the limit of detection. In R8, although they have not been quantified, SO_2_ and other inorganic pollutants of outdoor origin, such as NO_x_, O_3_, and marine aerosols (as measured at a station near the museum [28]), are likely to be present, because the visitors’ entrance door remains open for several hours a day.

### 3.2. Visual Changes and Colour Measurements

**Figure 3:**
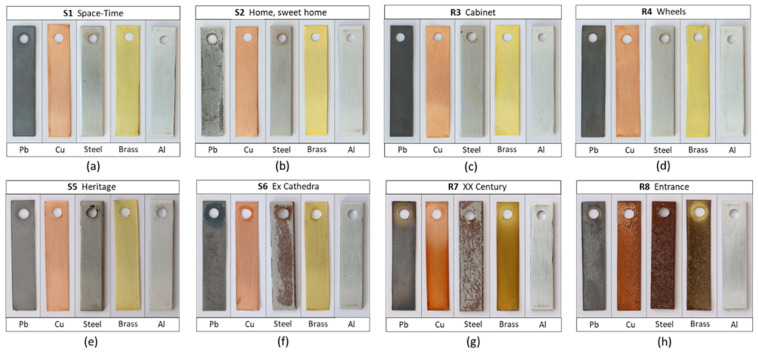
Metal coupons after 9 months of exposure in the eight locations of the MUNCYT museum. (**a**–**d**) correspond to showcases and rooms at the Alcobendas venue and (**e**–**h**) to the A Coruña venue.

The aesthetic changes that heritage objects undergo during their exhibition is an important issue, as they are an indication of degradation. Therefore, by visually inspecting the coupons on a macroscale (Figure 3) and taking into account the environmental data, a first approximation can be made as to which locations are most aggressive for the different metals, as carried out during the Oddy test [15,16]. In general, and at first glance, the coupons exhibited in showcases S5 and S6 (Figure 3e,f) and in rooms R7 and R8 (Figure 3g,h) at MUNCYT A Coruña appear to have suffered more changes than those in Alcobendas. This is mostly due to the humidity problems at the venue, which are also demonstrated by the corrosion of the steel (a metal that is mainly sensitive to high humidities and chlorides), especially in S6, R7 and R8. In these three locations, major changes are also observed in copper and brass coupons, and especially in all metal coupons in R8; this is due to the contribution of the outdoor environment. The appearance of white corrosion products on lead is a strong indicator of the presence of organic acids, which are characterised in Table 2. In S5, and above all, in S2, which showed the highest HCOOH content, lead coupons have a whitish and powdery appearance (Figure 3b) [29].

**Figure 4:**
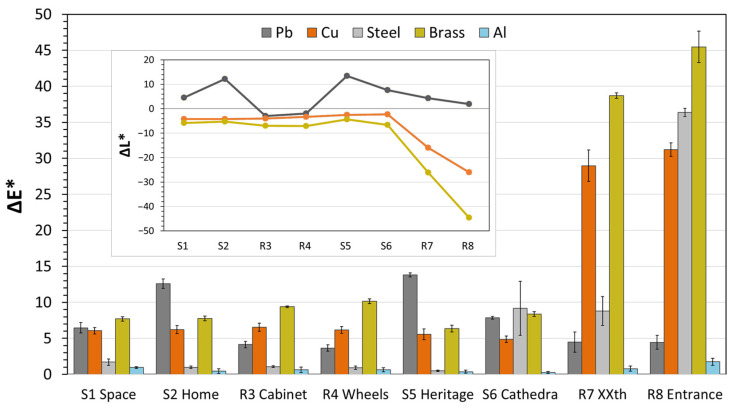
Colour differences between coupons after exposure. An inset plot for the difference in luminosity is included. Bars are the average of the three coupons for each metal with its SD.

The chromatic coordinates L*a*b* of the coupons after exposure and determination of the colour difference (∆E), as shown in Figure 4, allow us to quantify the observed visual changes. The metal with the least colour change is aluminium, with negligible change observed in all locations except R8, followed by the steel, exposed in all locations at Alcobendas and S5 at A Coruña, which showed ∆E <3 units, being imperceptible to the naked eye (Figure 4). For copper and brass (which seemed visually unchanged at most of the locations (Figure 3)), the colour differences range from 5 to 10 units, with the exception of R7 and R8, where this result was tripled. In both cases, visible corrosion occurred to a lesser or greater extent. The major contribution to the colour differences in these two metals and in lead is made by the difference in luminosity (∆L*), as can be seen in the inset plot of Figure 4. Brass tends to darken more than copper, and lead tends to whiten in some locations and darken in others; this can be explained by the different natures of the corrosion products formed.

### 3.3. Corrosion Rate by Mass Increase

**Figure 5:**
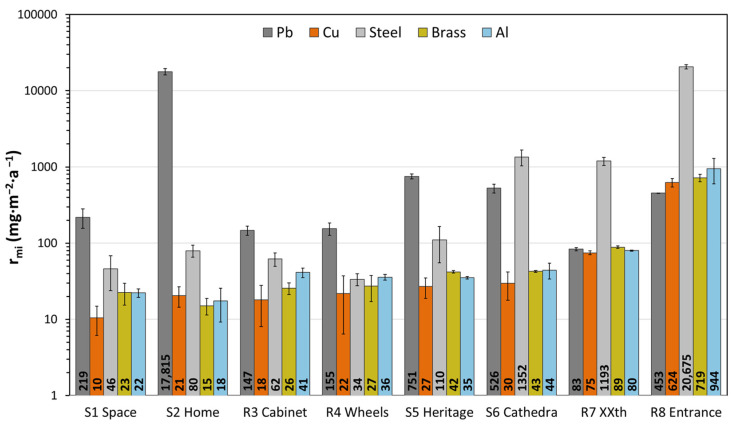
Corrosion rate of coupons based on mass increase. The data represented are the average of the three coupons for each metal and its respective error bars (SD).

All coupons gained mass after exposure, particularly those at location R8 (Figure 5), where there was particulate deposition of marine aerosols (see Section 3.4), (wherein chloride-containing products are identified), has greatly influenced the corrosivity of the environment. Comparing the *r_mi_* values obtained with the colour differences ∆E in Figure 4, the same trend seems to be followed between coupons of the same metal.

Lead has the highest *r_mi_,* and shows that the ratio is higher in coupons with the higher colour difference and luminosity, due to the formation of white lead compounds such as carbonates, acetates or formates [12], in locations with higher concentrations of organic acids (S2 and S5, and to a minor extent S6); the lowest values of *r_mi_* and ∆L* are related to the first corrosion products formed, such as lead oxides. Since weight gain is actually measured as the mass increase due to the anions that have reacted with the metal dissolved in the anodic corrosion reaction, it is more sensible to the formation of high molecular weight corrosion products (such as carbonates) than to oxides. Thus, a direct comparison of metallic lead loss is not possible, but from the point of view of conservation, oxides are stable and do not compromise the conservation of the object. Hence, both a large colour change towards lighter colours and a large increase in mass is an indication of poor conservation conditions for lead. This formation of the oxide layer may also be the cause of the mass increase in aluminium, copper and brass, although for the low weight gains observed in most locations, the contribution of surface deposits cannot be disregarded. In the case of aluminium, it is remarkable that in R8, the mass gain is more than one order of magnitude higher than in the other locations. Considering also that the typical corrosion morphology of aluminium is localized (and not uniform, as in the other metals studied), this high mass increase indicates a severe deterioration in R8. For the copper and brass, it can be appreciated that as *r_mi_* increases, so does ∆L*, in the negative direction (Figure 4; inset plot), as the coupons become more reddish [30] and ochre, respectively. For copper exposed to formic acid environments, since the cuprite layer formed is very thin, colour measurements have proven to be more sensitive and to have less dispersion than weight gain measurements [12]. The steel coupons are more difficult to correlate, as the corrosion products formed are very heterogeneous; however, it can be observed that steel is the second metal with the highest rate of mass increase.

Only copper and steel can be categorised when the corrosivity classification of ISO 11844-1 is considered [18]. For lead and brass, a classification is proposed based on the results obtained in the previous sections (Table 3). The classification of lead according to mass gain is complicated by the fact that the corrosion products formed are poorly adherent, and by the aforementioned effect of the nature of the corrosion product formed. For this study, where only in S2, lead is identified in a powdery state (Figure 3b) and *r_mi_* is very high relative to the other coupons (17,815 mg·m^−2^·a^−1^), the highest classification (IC 5) could be assigned. The rest of the lead coupons are classified by taking into account that the lowest categories are assigned to coupons that have experienced lower *r_mi_* and a darkening that is associated with the most stable and adherent corrosion, possibly lead oxide. To classify brass, the same values of the standard as for copper (or an average between copper and zinc) could be used, since the coupons obtained very similar results in all environments in terms of colour and mass increase.

From this classification, it can be determined that for copper and brass, the corrosivity of all locations is low or very low (with the exception of R8), possibly due to the low presence of sulphides, to which they are more susceptible [6,12]. For steel, the corrosivity is strongly dependent on the relative humidity, with a lower effect of typical indoor pollutants. At the MUNCYT A Coruña locations, this is an issue because even in showcases, the corrosivity is classified as medium (Table 3; S6). The presence of organic acids makes the corrosivity classification very unfavourable for lead, so attention must be paid to the exposure of artefacts containing this metal. Regarding aluminium, the rate of mass increase is not enough to propose a classification, but it is proposed that the corrosivity may be low—with the exception of R8—due to the oxide layer that may have formed during exposure.

### 3.4. Corrosion Product Characterisation

**Figure 6:**
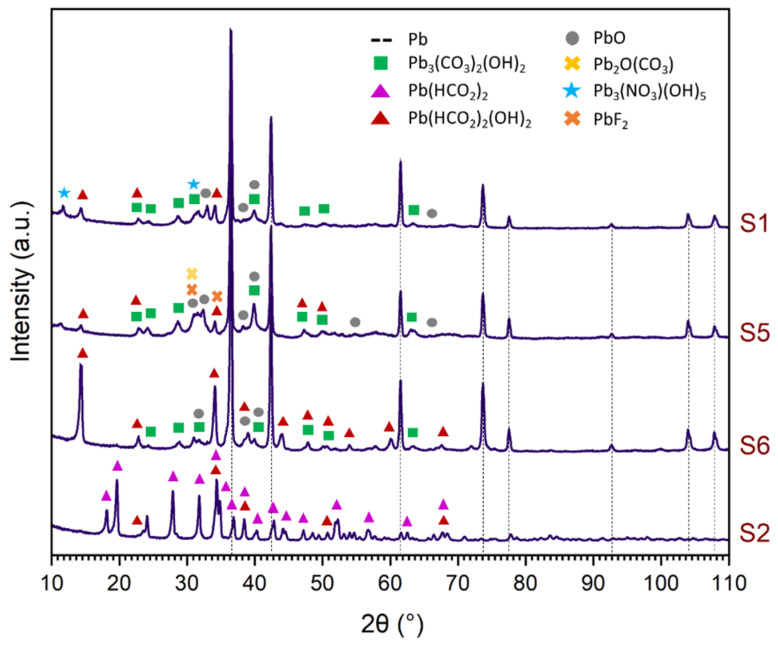
Identification of corrosion products formed on lead in the showcases of MUNCYT Alcobendas (S1, S2) and MUNCYT A Coruña (S5, S6).

XRD characterisation of the corrosion products formed in the metallic dosimeters during exposure allows us to make a comparison with the results obtained so far, and to evaluate to what extent the corrosion formed may be more or less harmful for the conservation of each metal. Since the highest concentration of pollutants, especially in showcases, has been of formic and acetic acids, lead has been fully characterised by its sensitivity and the higher formation of corrosion products (Figure 6). When coupons are exposed to acetic and formic acids, the pollutants react with the oxide layer, forming powdery deposits of lead acetates and formates. In the presence of atmospheric carbon dioxide and humidity, these organic salts react, forming lead carbonates and releasing acids that are available to further corrode the metallic lead [29]. With the high presence of formic acid in S1, S2, S5 and S6, soluble salts develop, and can be identified as lead formate (Pb(HCO_2_)_2_), formate hydroxide (Pb(HCO_2_)_2_(OH)_2_) and lead nitrate hydroxide (Pb_3_(NO_3_)(OH)_5_) (Figure 6). In S2, it is determined that the powdery state (Figure 3b) is particularly due to high formate formation (Figure 6), and as a result, corrosion is especially active. In S1, S5 and S6, corrosion is less aggressive, because carbonates predominate. The presence of lead fluoride is also determined in S5, which is related to the concentration of 0.14 µg/m^3^ HF obtained in this case (Table 2). In rooms, lead oxides predominate, followed by carbonates such as cerussite and hydrocerussite (Figure S1).

In copper and brass, the possible thin and very adherent layer of oxide which may have been responsible for the increase in the mass of the coupons, could not be characterised due to its low thickness, except for R7 and R8. Although in R7, the concentration of organic acids was not very high (3.7 µg/m^3^ formic and <2 µg/m^3^ acetic), the high RH of the room (70% on average) led to the formation of a more visible and detectable cuprite layer [12] (Figure S2). The same has occurred with brass, wherein zinc oxide is identified (Figure S3).

The more voluminous corrosion products have been identified in R8, where it is determined that pollutants from outside contributed significantly. In addition to a high and difficult-to-control RH at this location, the entrance of marine aerosols bearing chloride ions (Cl^−^) is demonstrated by the identification of akaganeite (Fe^3+^O(OH)) in steel, and botallackite (Cu_2_(OH)_3_Cl) and zinc oxide chloride hydrate in brass (Figures S4 and S5, respectively). Aluminium is the metal that changed the least in all locations, due to the protective passivation layer of aluminium oxyhydroxides that it forms [29]. However, in the presence of chlorides and high RH, this passivation layer starts to rupture, thereby developing pitting corrosion. Additionally, 2000-series alloys, such as the 2024 used in this study, are especially prone to this type of corrosion due to the precipitation of intermetallic particles such as Al_7_Cu_2_Fe and Al_2_Cu present in the alloy, which compromise the resistance of the passivation layer [31] (Figure S6).

### 3.5. Final Considerations

Thanks to the indoor environment assessment carried out, and our understanding of how the presence of pollutants can affect the different metals that are present in the ST collection, it is possible to identify the most problematic locations for the conservation of these artefacts. In general, in Alcobendas, the environmental conditions of the locations are more controlled, and the metals of the objects that are exhibited directly in rooms (such as R4) have a lower risk of corrosion than those in the showcases, due to ventilation. This is not the case if the room has unfavourable environmental conditions, and is even worse in the presence of contaminants from outside, e.g., in R7 and R8. This last location is the most problematic and corrosive, and the exhibition of objects therein, such as the aluminium aircraft, is not advisable due to the corrosion problems suffered by the coupons.

Regardless of the venue, the presence of contaminants that can damage the objects to a greater or lesser extent has been determined in all the showcases. S1 is the showcase with the greatest variety of materials that could be a source of emissions; however, as it is several metres long and the objects are exposed far apart, it was not identified as problematic. This is not the case with S2, wherein many objects with wood and plastics—as the main source of emissions—[14] are displayed in a smaller space; therefore, it is not advisable to exhibit objects that corrode easily in the presence of organic acids. The same applies to showcase S5, wherein the material emitting organic acids and sulphides could be leather [32] (on the case of an astrological disc) or wood (of the three artefacts exhibited).

In addition to the typical degradation products caused by well-known indoor and outdoor pollutants, more exotic corrosion products have been identified, such as lead nitrates and fluorides (in a location where HF has also been detected). Both have been found inside display cases, and the fact that other display cases made with the same materials do not present this problem points to the materials of the artefacts being the source of the pollutants. The exact origin of the pollutants has not been identified, but this is a good example of the challenges to conservation that are posed by the wide variety of materials making up ST collections.

## 4. Conclusions

The use of different methods to evaluate, classify, and characterise the corrosion suffered by the metals studied allows for a better understanding of how environmental and exposure conditions can contribute to the degradation of scientific and technical artefacts. Considering the complexity of these types of collections due to their wide material variety, keeping the relative humidity and temperature conditions low has proven to be of great importance in preventing, as far as possible, the metal part of the objects from corrosion. At the A Coruña venue, it is recommended to check the climate control system and to keep the relative humidity at low levels.

By identifying, in particular, the typical contaminants inside the showcases, first decisions can be made as to which metals should or should not be exposed in that environment. Additionally, by characterising the variation in the mass, colour and corrosion products formed, the level of corrosion damage can be determined.

In cases in which a high concentration of organic acids or other compounds such as hydrogen fluoride have been detected as contributing to lead corrosion, further studies should be carried out to specifically identify the source of the emission. This requires characterisation of the materials co-existing at the same location, and an Oddy test for those suspected of being corrosive. Moreover, in cases in which it is not possible to control these pollutants and environmental and exposure conditions of the artefacts, remedial conservation measures, such as applying protective coatings that act as a barrier to moisture and contaminants, may be a good solution. 

## Figures and Tables

**Table 1:** Classification of corrosivity of indoor environment using the rate of mass increase of steel, copper, and zinc according to [18].

Corrosivity Category		Rate of Mass Increase (*r_mi_*) (mg·m^−2^·a^−1^)		
		Steel	Zinc	Copper
IC 1	Very low	*r_mi_* ≤ 70	*r_mi_* ≤ 50	*r_mi_* ≤ 25
IC 2	Low	70 < *r_mi_* ≤ 700	50 < *r_mi_* ≤ 250	25 < *r_mi_* ≤ 100
IC 3	Medium	700 < *r_mi_* ≤ 7000	250 < *r_mi_* ≤ 700	100 < *r_mi_* ≤ 450
IC 4	High	7000 < *r_mi_* ≤ 50,000	700 < *r_mi_* ≤ 2500	450 < *r_mi_* ≤ 1000
IC 5	Very high	50,000 < *r_mi_* ≤ 150,000	2500 < *r_mi_* ≤ 5000	1000 < *r_mi_* ≤ 2500

**Table 2:** Ambient conditions (humidity and temperature) and pollutant concentrations measured at the different locations.

	Ambient Conditions						Pollutant Concentration (µg/m^3^) According to STP *^1^					
Locations	T (°C) Average	T_max_	T_min_	RH (%) Average	RH_max_	RH_min_	SO_2_	HF	HCOOH	CH_3_COOH	HCl	H_2_S
S1	23.4	26.5	21.0	35.5	52.5	19.5	<0.1	<0.1	54	<2.0	<0.3	5.3
S2	21.4	27.3	19.2	39.4	49.5	30.5	<0.1	<0.1	>200 *^2^	3.5	<0.3	2.7
R4	21.2	27.2	17.9	42.6	65.9	22.4	<0.1	<0.1	8.3	<2.0	<0.3	2.6
S5	20.7	27.0	16.0	52.7	62.0	37.5	<0.1	0.14	>200	107	<0.3	7.0
S6	20.5	27.0	16.5	55.9	66.0	37.5	<0.1	<0.1	63	97	<0.3	1.8
R7	22.2	30.6	16.6	70.6	91.8	48.4	0.22	<0.1	3.7	<2.0	<0.3	2.8

*^1^ STP = Standard temperature 20 °C and pressure 1013 hPa. *^2^ <Below detection limit.> Above detection limit.

**Table 3:** Classification of the corrosiveness of the indoor environment of museum locations according to the corrosion rate of mass increase.

	Steel (ISO 11844)	Cu (ISO 11844)	Pb (Proposal)	Brass (Proposal)
S1 Space	IC 1	IC 1	IC 2	IC 1
S2 Home	IC 2	IC 1	IC 5	IC 1
R3 Cabinet	IC 1	IC 1	IC 2	IC 1
R4 Wheels	IC 1	IC 1	IC 2	IC 1
S5 Heritage	IC 2	IC 2	IC 4	IC 2
S6 Cathedra	IC 3	IC 2	IC 3	IC 2
R7 XXth	IC 3	IC 2	IC 2	IC 2
R8 Entrance	IC 4	IC 4	IC 3	IC 4

## Data Availability

The datasets generated and analysed during the current study will be available in the following institutional repository: https://digital.csic.es/ (accessed on 7 June 2023).

## Supplementary Materials

The following supporting information can be downloaded at: https://www.mdpi.com/article/10.3390/ma16124239/s1. Figure S1: Characterisation via XRD of the corrosion products formed on lead coupons in room R4, “Wheels”, of MUNCYT Alcobendas; Figure S2: Characterisation via XRD of the corrosion products formed on copper coupons in room R7, “XX Century”, of MUNCYT A Coruña; Figure S3: Characterisation via XRD of the corrosion products formed on brass coupons in room R7, “XX Century”, of MUNCYT A Coruña; Figure S4: Characterisation via XRD of the corrosion products formed on steel coupons of room R8, “Entrance”, of MUNCYT A Coruña; Figure S5: Characterisation via XRD of the corrosion products formed on brass coupons in room R8, “Entrance”, of MUNCYT A Coruña; Figure S6: Characterisation via XRD of the corrosion products formed on aluminium coupons in room R8, “Entrance”, of MUNCYT A Coruña.
