# Supplementary material for: Corrosion Risk to Metal-Based Artefacts in a Scientific and Technical Museum: An Assessment of Environmental and Exhibition Conditions
Source: Materials (Basel). 2023 Jun 8;16(12):4239. doi: 10.3390/ma16124239 (PMC10302543; doi:10.3390/ma16124239)
Supplement: Supplementary file 1 [file materials-16-04239-s001.zip › materials-2399071-supplementary.pdf]

materials

Supplementary Materials

Corrosion Risk to Metal-Based Artefacts in a Scientific and Technical Museum: An Assessment of Environmental and Exhibition Conditions

María Teresa Molina *, Emilio Cano, Irene Llorente and Blanca Ramírez-Barat

Centro Nacional de Investigaciones Metalúrgicas (CENIM), Consejo Superior de Investigaciones Científicas (CSIC), 28040 Madrid, Spain
* Correspondence: mt.molina@cenim.csic.es

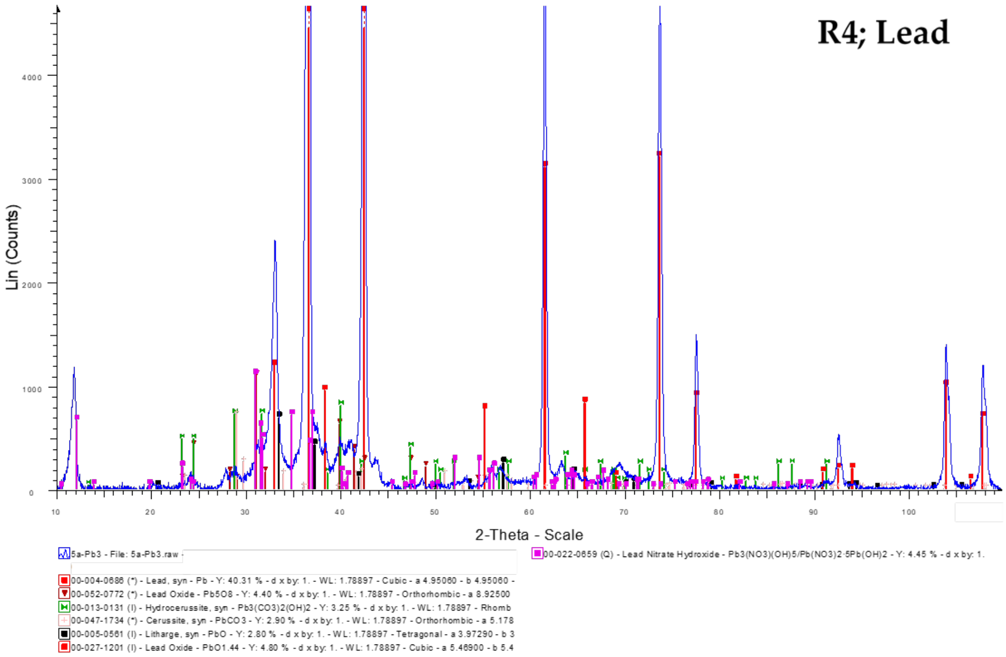

Figure S1. Characterisation via XRD of the corrosion products formed on lead coupons in room R4, "Wheels", of MUNCYT Alcobendas.

Citation: Molina, M.T.; Cano, E.; Llorente, I.; Ramírez-Barat, B. Corrosion Risk to Metal-Based Artefacts in a Scientific and Technical Museum: An Assessment of Environmental and Exhibition Conditions. *Materials* **2023**, *16*, 4239. https://doi.org/10.3390/ma16124239

Academic Editor: Xin-Yun Wang

Received: 28 April 2023
Revised: 1 June 2023
Accepted: 6 June 2023
Published: 8 June 2023

Copyright: © 2023 by the authors. Licensee MDPI, Basel, Switzerland. This article is an open access article distributed under the terms and conditions of the Creative Commons Attribution (CC BY) license (https://creativecommons.org/licenses/by/4.0/).

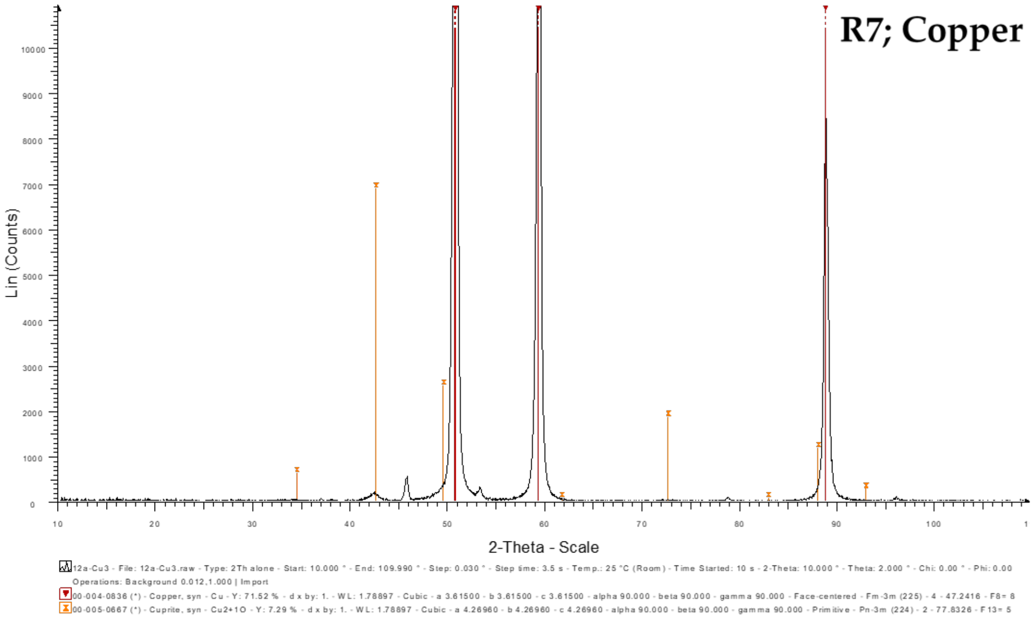

Figure S2. Characterisation via XRD of the corrosion products formed on copper coupons in room R7, "XX Century", of MUNCYT A Coruña.

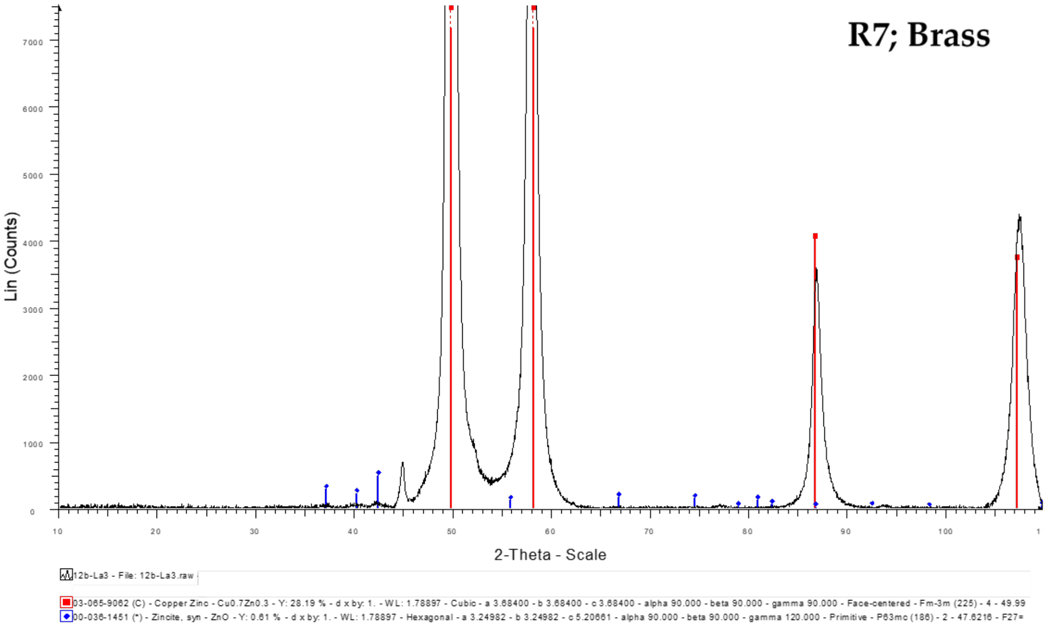

Figure S3. Characterisation via XRD of the corrosion products formed on brass coupons in room R7, "XX Century", of MUNCYT A Coruña.

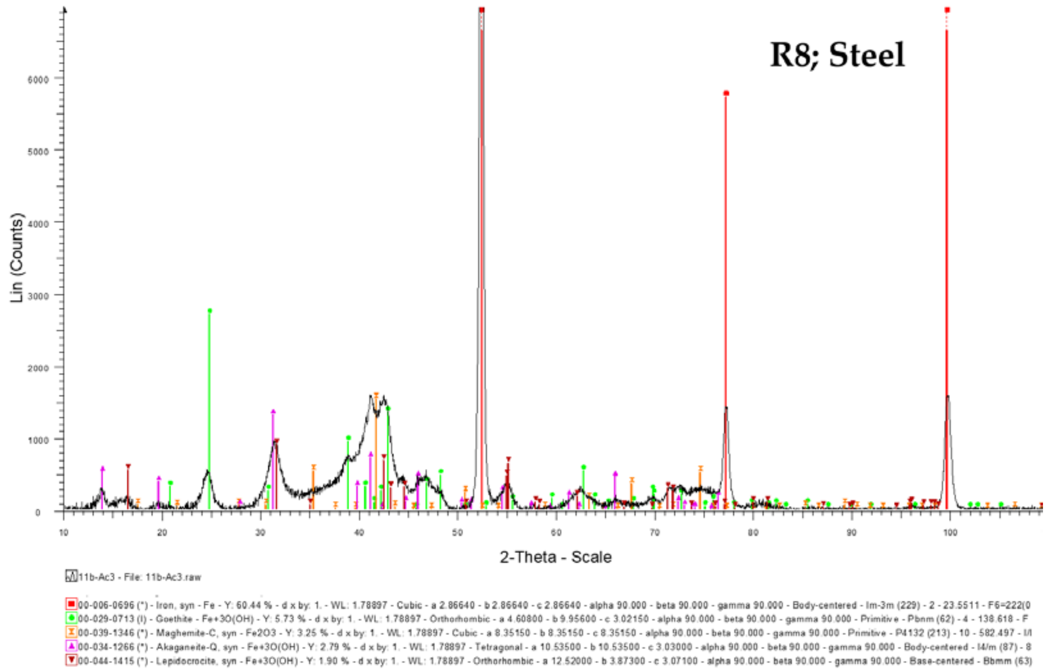

Figure S4. Characterisation via XRD of the corrosion products formed on steel coupons of room R8, “Entrance”, of MUNCYT A Coruña.

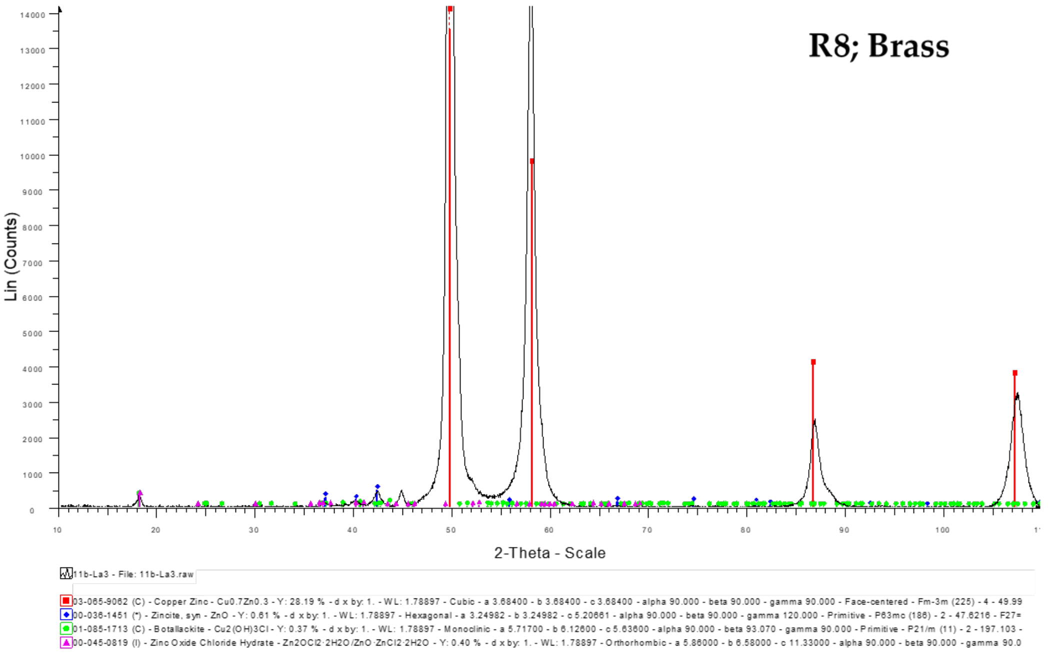

Figure S5. Characterisation via XRD of the corrosion products formed on brass coupons in room R8, “Entrance”, of MUNCYT A Coruña.

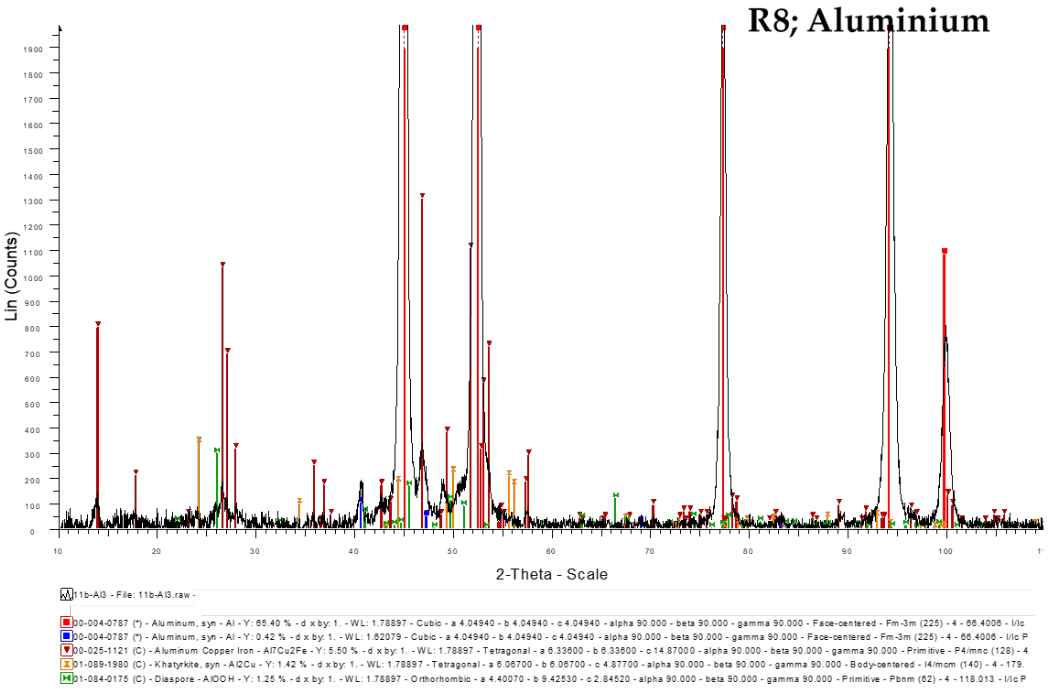

Figure S6. Characterisation via XRD of the corrosion products formed on aluminium coupons in room R8, “Entrance”, of MUNCYT A Coruña.
